# High levels of ubidecarenone (oxidized CoQ_10_) delivered using a drug-lipid conjugate nanodispersion (BPM31510) differentially affect redox status and growth in malignant glioma versus non-tumor cells

**DOI:** 10.1038/s41598-020-70969-0

**Published:** 2020-08-17

**Authors:** Jiaxin Sun, Chirag B. Patel, Taichang Jang, Milton Merchant, Chen Chen, Shiva Kazerounian, Anne R. Diers, Michael A. Kiebish, Vivek K. Vishnudas, Stephane Gesta, Rangaprasad Sarangarajan, Niven R. Narain, Seema Nagpal, Lawrence Recht

**Affiliations:** 1 Department of Neurology and Clinical Neurosciences, Stanford University, Palo Alto, CA 94305 USA; 2 Molecular Imaging Program at Stanford (MIPS), Department of Radiology, Stanford University School of Medicine, Stanford, CA 94305 USA; 3 Department of Otolaryngology, Stanford University, Palo Alto, CA 94305 USA; 4 BERG LLC, Framingham, MA 01701 USA

**Keywords:** Cancer metabolism, CNS cancer

## Abstract

Metabolic reprogramming in cancer cells, vs. non-cancer cells, elevates levels of reactive oxygen species (ROS) leading to higher oxidative stress. The elevated ROS levels suggest a vulnerability to excess prooxidant loads leading to selective cell death, a therapeutically exploitable difference. Co-enzyme Q_10_ (CoQ_10_) an endogenous mitochondrial resident molecule, plays an important role in mitochondrial redox homeostasis, membrane integrity, and energy production. BPM31510 is a lipid-drug conjugate nanodispersion specifically formulated for delivery of supraphysiological concentrations of ubidecarenone (oxidized CoQ_10_) to the cell and mitochondria, in both in vitro and in vivo model systems. In this study, we sought to investigate the therapeutic potential of ubidecarenone in the highly treatment-refractory glioblastoma. Rodent (C6) and human (U251) glioma cell lines, and non-tumor human astrocytes (HA) and rodent NIH3T3 fibroblast cell lines were utilized for experiments. Tumor cell lines exhibited a marked increase in sensitivity to ubidecarenone vs. non-tumor cell lines. Further, elevated mitochondrial superoxide production was noted in tumor cells vs. non-tumor cells hours before any changes in proliferation or the cell cycle could be detected. In vitro co-culture experiments show ubidecarenone differentially affecting tumor cells vs. non-tumor cells, resulting in an equilibrated culture. In vivo activity in a highly aggressive orthotopic C6 glioma model demonstrated a greater than 25% long-term survival rate. Based on these findings we conclude that high levels of ubidecarenone delivered using BPM31510 provide an effective therapeutic modality targeting cancer-specific modulation of redox mechanisms for anti-cancer effects.

## Introduction

The Warburg effect was originally described a century ago as an aspect of metabolic rewiring in cancer cells^1,2^, and is now considered a distinctive hallmark of cancer, emerging in recent years as an important concept in the field of cancer biology^3^. Further, recent studies reveal the Warburg phenotype as more than the simple overutilization of glycolysis vs. oxidative metabolism; rather, it reflects a complex re-circuitry of the metabolic machinery, culminating in the facilitation of a hyper-proliferative state^4,5^. The study of metabolic reprogramming in cancer highlights, as a potential vulnerability, the increased levels of steady state reactive oxygen species (ROS) relative to normal tissue^6–8^.

ROS, which include H_2_O_2_, superoxide anions (O_2_^−^), and hydroxyl radicals (OH^•^), are byproducts of aerobic metabolism, previously considered detrimental to cellular health, but now recognized as important signal transducers with optimal cellular function ranges, which if exceeded, induce pathology due to the increased oxidative stress that damages lipids, proteins, and DNA^9^. Metabolic reprogramming in cancer cells results in the generation of higher than normal levels of ROS from mitochondria and cytoplasmic NADPH oxidases^10,11^, which require counterbalancing through antioxidant activity^12^. Consequently, the elevated levels of ROS in cancer cells create a potential vulnerability to prooxidants, rendering them susceptible to oxidative-stress-induced cell death^13,14^. Conventional anti-cancer agents such as doxorubicin are in fact prooxidants that drive ROS levels above a death-inducing threshold in cancer cells^15–18^; however, due to toxicity, there are limits on dosing, emphasizing the need for less toxic agents with similar functions based on inducing selective ROS production. CoQ_10_ (ubidecarenone) is a lipophilic antioxidant with the potential to serve as the basis for the aforementioned strategy.

CoQ_10_ is hydrophobic due to its side chains, and thus resides in membranous fractions such as mitochondria and plasma membranes^19,20^, naturally serving as an electron carrier, exploiting the redox profile of the p-benzoquinone ring moiety^21,22^. Within the inner mitochondrial membrane, the activity of CoQ_10_ is dependent on its redox state^23^ of which there are three: oxidized (ubiquinone, also known as ubidecarenone or CoQ_10_), a free-radical intermediate (semiquinone, CoQ_10_H^•^), and the most abundant reduced form (ubiquinol, CoQ_10_H_2_)^24,25^.

In its reduced form, CoQ_10_H_2_ serves as a potent endogenous antioxidant that prevents lipid peroxidation, protein carbonylation, and oxidative damage to DNA^26^. The aforementioned antioxidant function is sub-served via two types of reducing quinone-related oxidoreductases. NADPH dehydrogenase (quinone) 1 catalyzes the two-electron reduction of quinones, producing stable quinols^27–29^. In contrast, enzymes such as NADPH-cytochrome P450 reductase catalyze the reduction to a semiquinone radical in the presence of a suitable electron donor such as NADPH^27,30,31^, which because of its own lability and high reactivity easily donates an electron to a neighboring oxygen molecule, resulting in the production of an O_2_^−^ anion. Multiple such reactions result in an overabundance of O_2_^−^ anions and consequently cell toxicity. Considering the increased levels of oxidative stress within cancer cells, exposure to the optimum amount of CoQ_10_ could potentially exclusively affect cancer cells, thus providing a potentially well-tolerated and effective anti-cancer therapy.

The aforementioned approach is however limited by CoQ_10_′s insolubility, which restricts the amount that can be delivered to cells, and to date, only modest anti-cancer efficacy has been reported^32^. Furthermore, since oxidative stress can be cell supportive when therapeutic agents fail to raise ROS levels beyond toxic thresholds^33^, the potential for a cancer therapeutic agent to work will depend on its markedly increased delivery to cancer tissues.

To address the aforementioned challenge, an oxidized form of CoQ_10_ (ubidecarenone) formulated as a drug-lipid conjugate nanodispersion (BPM31510) and optimized for stability and delivery was developed^34^ to investigate the therapeutic potential of delivering supraphysiological concentrations of ubidecarenone to tumors. Given that CoQ_10_ is known to cross the blood brain barrier^35,36^, we investigated its efficacy in the treatment-refractory malignant glioma. Using co-cultures of glioma cells and non-tumor cells, we demonstrate that BPM31510 treatment differentially and rapidly raises intramitochondrial O_2_^−^ anion levels in glioma cells relative to non-tumor cells, an effect that precedes any changes in proliferation or cell cycle status. Importantly, we demonstrate unique in vivo activity using an orthotopic glioblastoma model. These findings suggest a selective therapeutic potential for BPM31510 in the highly aggressive glioma cancer cells with minimal impact on non-tumor cells.

## Results

### Differential effects of ubidecarenone on glioblastoma and non-tumor cell lines

**Figure 1:**
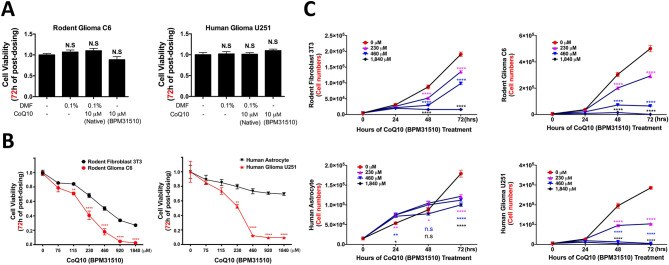
Differential effects of oxidized CoQ_10_ on glioblastoma and non-tumor cell lines. (**A**) Relative cell viability of rodent C6 glioma and human U251 glioma cells after exposure to 0.1% DMF, 10 µM native CoQ10 (in 0.1% DMF), or 10 µM of ubidecarenone using BPM31510 for 72 h. Values are normalized to control. No significant effect on cell growth is noted under any condition. (**B**) Dose response curves for rat C6 glioma and mouse NIH3T3 fibroblast cells (left panel) and human U251 glioma and HA cells (right panel) after incubation for 72 h with BPM31510. Note that in each case, the tumor line was more sensitive than the control. (**C**) Cell viability analysis of each cell line over time after incubation with increasing doses of ubidecarenone. The number of live cells is converted from a pre-established standard curve with known cells numbers. All data presented as Mean ± SEM. *P < 0.05, **P < 0.01, ***P < 0.001, ****P < 0.0001 compared to control (no BPM31510) counts on the same day. Each color corresponds to the BPM31510 dose.

CoQ_10_ is a highly lipophilic molecule with limited water solubility that requires dissolving in highly toxic organic solvents such as ethanol (0.3 mg/ml) or dimethyl formamide (DMF, 10 mg/ml) prior to use. Based on preliminary studies, we determined that a limit of 0.5% DMF in cell cultures prevented solvent toxicity, thus limiting the concentration of CoQ_10_ to a maximum of 10 µM (Supplementary Figure S1). At this dose, neither native ubidecarenone nor BPM31510-delivered ubidecarenone had an effect on cell proliferation in either the rat C6 or human U251 glioma cells (Fig. 1A).

The lipid formulation of BPM31510 enables the achievement of supraphysiological ubidecarenone levels allowing for assessment of much higher dosing. Figure 1B demonstrates the effects of higher ubidecarenone dosing on the cell viability of two glioma cell lines, rat C6 and human U251, and two non-tumor cell lines, mouse NIH3T3 fibroblast cells (immortalized but not neoplastic) and normal human astrocytes (HA). After 72 h of incubation, rat C6 glioma cell growth was inhibited to a significantly higher degree than mouse NIH3T3 cell growth (IC_50_ C6: 230 μM vs. IC_50_ NIH3T3: > 460 μM). Interestingly, a more dramatic effect was observed in the human U251 glioma and HA cells (IC_50_ U251: 230 µM vs. IC_50_ HA: 1,840 µM), suggesting that the non-tumor HA cells were essentially unaffected by the high concentrations of ubidecarenone (Fig. 1B).

To assess growth inhibitory effects of ubidecarenone on glioma and non-tumor cells, timed proliferation assays were performed as a function of drug dose; differential effects were noted over time (Fig. 1C). Notably, a cytostatic effect of ubidecarenone was noted at the lower doses (230 µM and 460 µM) upon treatment of rat C6 glioma cells, while a cytocidal effect was noted at the highest dose (1,840 µM). Similar but more pronounced results were noted for human U251 glioma cells, where both 460 µM and 1,840 µM doses were cytocidal. In contrast, only cytostatic effects were noted for mouse NIH3T3 fibroblast cells, and the highest dose of BPM31510 had minimal effect on the non-tumor HA cells. In fact, a sixfold increase in HA cell numbers was observed after a 72-h exposure to 1,840 µM of Ubidecarenone, suggesting maintenance of proliferative responses for these non-tumor cells.

### Ubidecarenone induces G_2_/M cell cycle arrest of glioblastoma but not non-tumor cell lines

**Figure 2:**
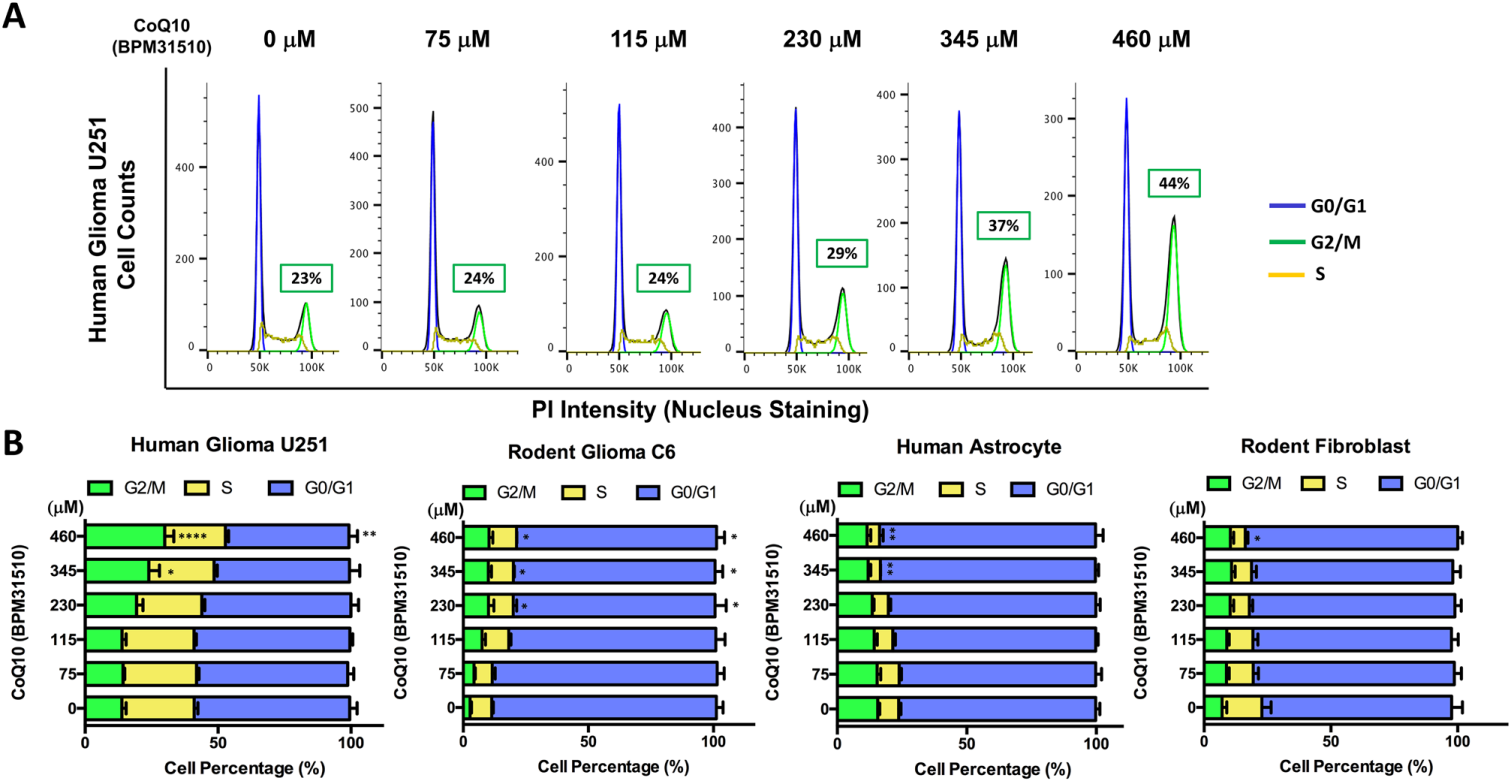
Ubidecarenone induces G_2_/M cell-cycle arrest of glioblastoma but not non-cancerous cell lines. (**A**) Cell cycle analysis and quantification of human U251 glioma cells treated with 0, 75, 115, 230, 345, or 460 µM ubidecarenone. The percentage of cells in each phase (G_0/1_, S or G_2_ + M) is estimated from the frequency histograms. The percentage of cells in G_2_ + M phase is highlighted. (**B**) Quantification of human U251 glioma, rat C6 glioma, HA, or mouse NIH3T3 fibroblast cells in each cell phase. All data presented as mean ± SEM. *P < 0.05, **P < 0.01, ***P < 0.001, ****P < 0.0001 compared to control (no drug exposure) at the same cell cycle phase.

To define the mechanistic underpinnings of ubidecarenone’s growth inhibitory effects, we conducted cell cycle analysis. As demonstrated in Fig. 2A, treatment of human U251 glioma cells with 460 µM ubidecarenone for 48 h resulted in significant accumulation of cells in the G2/M phase. We next performed cell cycle analyses for both glioma and non-tumor cell lines at various doses (Fig. 2B). In the glioma cells, a significant dose-dependent relationship with regards to G2/M phase arrest was noted, while in the non-tumor cells, the differences noted at all doses were not statistically significant. Of note, the G2/M delay was significant in both glioma cell lines at approximately the IC_50_ dose (between 230 µM and 460 µM ubidecarenone).

### Differential redox vulnerabilities to ubidecarenone exposure between glioma and non-tumor cells

**Figure 3:**
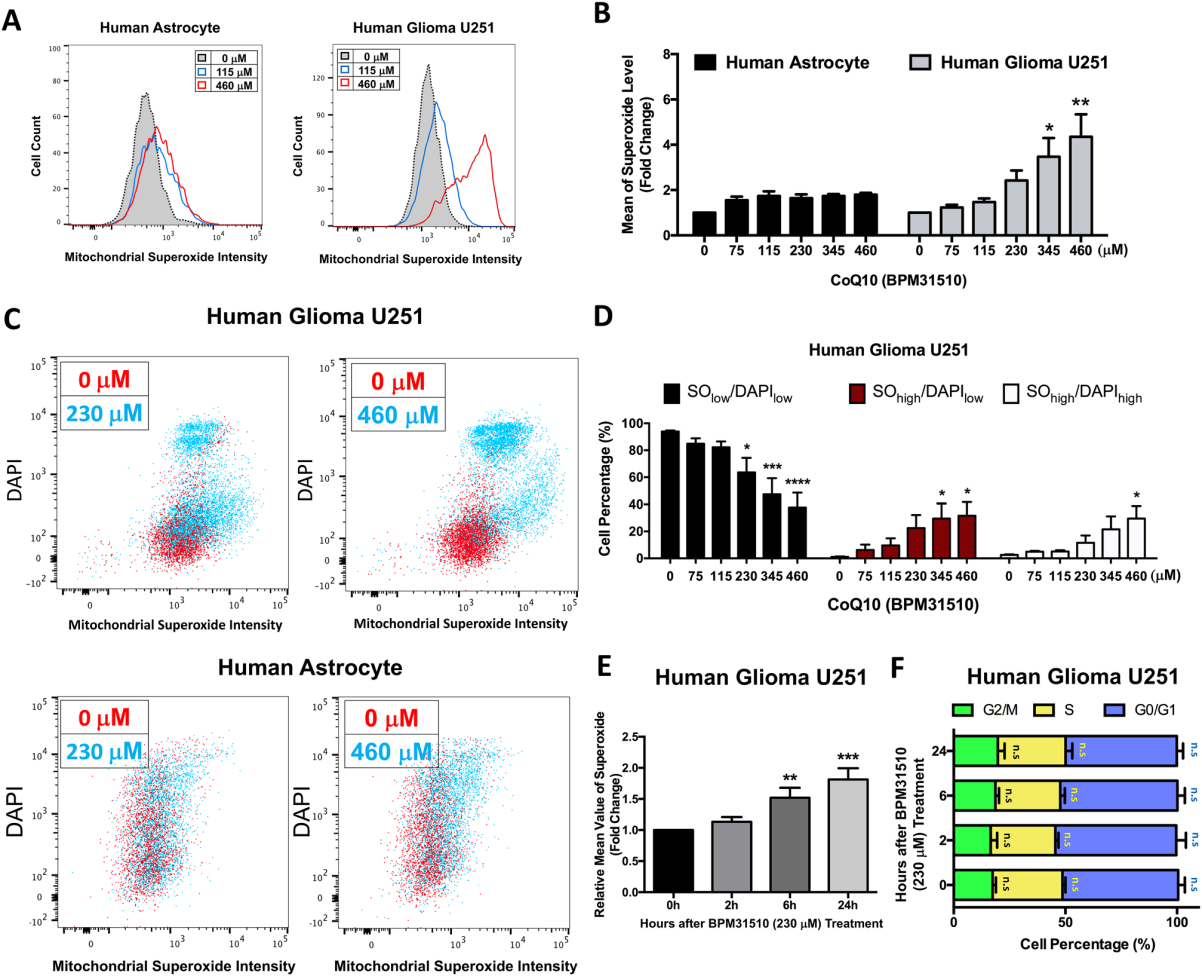
Differential redox vulnerabilities to ubidecarenone exposure between non-tumor and glioblastoma cells. (**A**) Flow cytometry analysis of O_2_^−^ in HA or human U251 glioma cells treated with 0, 115, or 460 µM ubidecarenone. O_2_^-^ intensity in each cell is demonstrated in the frequency histograms. (**B**) Quantification of mean O_2_^−^ levels in HA and human U251 glioma cells. A modest increase in O_2_^−^ is noted in HA in the presence of ubidecarenone, although this increase is not dose-dependent. In contrast, there is a fourfold O_2_^−^ increase in rat C6 glioma cells in a dose-dependent manner. (**C**) Flow cytometry analysis (scatter plot) of O_2_^−^ and DAPI in human U251 glioma and HA cells. Notably, there is minimal change in O_2_^−^ intensity with increasing ubidecarenone dose in HA cells, while there is a marked increase in both O_2_^−^ and DAPI in human U251 glioma cells after drug exposure. (**D**) Quantification of relative cell populations of O_2_^−^_low_/DAPI_low_, O_2_^−^_high_/DAPI_low_, and O_2_^−^_high_/DAPI_high_ in human U251 glioma cells demonstrating increasing numbers of high O_2_^-^ and DAPI labeled cells with increasing ubidecarenone dose. **(E**) Flow cytometry analysis of O_2_^−^ for human U251 glioma cells treated with 230 µM ubidecarenone for 0, 2, 6, or 24 h. Relative mean values of O_2_^−^ were quantified and normalized to control at 0 h. (**F**) Cell cycle analysis by flow cytometry for human U251 glioma cells treated with 230 µM ubidecarenone for 0, 2, 6, or 24 h. The percentage of human U251 glioma cells in each cell cycle phase (G_0/1_, S or G_2_ + M) was quantified. All data presented as Mean ± SEM. *P < 0.05, **P < 0.01, ***P < 0.001, ns = not significant, compared to control (0 µM) at the same cell cycle phase.

Given that most ROS is produced within the mitochondria^37^, we next assessed differential effects of ubidecarenone on mitochondria-derived ROS by measuring O_2_^−^ production using a dye that specifically localizes within mitochondria. A 48-h incubation of human U251 glioma or HA cells with ubidecarenone concentrations between 0–460 µM resulted in a dose-dependent accumulation of O_2_^−^ that was markedly increased in the glioma cells as compared to HA cells (Fig. 3A,B). Specifically, we noted that while O_2_^−^ levels for HA cells were elevated approximately 1.8-fold when exposed to 115 µM ubidecarenone, higher doses did not result in additional increased ROS production. In contrast, a dose-dependent increase in O_2_^−^ production was noted in human U251 glioma cells, with the highest dose producing over a fourfold elevation in O_2_^−^ (Fig. 3B; Supplementary Fig. 2).

Additionally, DAPI co-staining was utilized to determine the flow cytometry profiles of human U251 glioma and HA cells. Human U251 glioma cells exhibited three distinct populations after exposure to BPM31510: O_2_^−^_low_/DAPI_low_, O_2_^−^_high_/DAPI_low_, and O_2_^−^_high_/DAPI_high_ (Fig. 3C and Supplementary Figure S3). In Fig. 3D, a dose-dependent accumulation of cells in both O_2_^−^_high_ and DAPI_high_ populations is graphically depicted. As shown in Fig. 3C (bottom panels) HA cell populations were indistinguishable even at the highest ubidecarenone dose. These data support the existence of differential redox vulnerabilities between glioma and non-tumor cells. Similar results were noted with the rat C6 glioma and mouse NIH3T3 cells, where images demonstrate the active superoxide (red) being highly correlated with the C6 (GFP) population, while essentially no fluorescent signal was noted until its occasional appearance with these nonfluorescent cells was noted at the highest doses examined (345 μM or 460 μM) (indicated by white arrows in Supplementary Figure S4). These observations affirm the differential induction of mitochondrial superoxide by BPM31510 in non-cancer vs. neoplastic cells.

### Ubidecarenone induces an early-onset and increased accumulation of O_2_^−^ that precedes cell cycle arrest

Given the noted increase in superoxide production and the decrease in proliferation after 48 h, we wanted to determine the sequence of the onset of cell cycle changes and ROS production in glioma and non-tumor cells. Consequently, we assessed both O_2_^−^ levels and cell cycle state in human U251 glioma cells incubated with 230 µM BPM31510 for 0, 2, 6, and 24 h. O_2_^−^ levels were significantly elevated after 6 h and continued to increase over the 24-h course of the experiment (Fig. 3E). In contrast, no significant changes were detected in cell cycle state even at 24 h (Fig. 3F). This supports the contention that elevated mitochondrial O_2_^−^ levels represent an early event in the cascade leading to slowed proliferation and death of cancer cells.

### Ubidecarenone differentially affects glioma and non-tumor cell growth in co-culture experiments

Given the higher levels of oxidative stress in cancer cells relative to non-cancer cells, a potential exists for capitalizing on these differences by differentially stressing the cancer cells through exposure to prooxidants. Therefore, we next assessed the effects of ubidecarenone on viability and redox homeostasis in both rodent and human co-cultures of glioma and non-tumor cells (Table 1).

**Table 1:** Strategy of human and rodent co-culture experiments using glioma and non-tumor cells.

Co-culture	Human		Rodent	
Predominant type of CoQ	CoQ10		CoQ9	

Cell type	Astrocyte	Glioma U251	Fibroblast NIH3T3	Glioma C6
Label	None	GFP	None	GFP
Starting ratio in population	60%	40%	50%	50%

In the human cells model, HA (non-labeled) and U251 glioma (GFP-labeled) cells are co-cultured at designated cell densities. In the rodent cells model, NIH3T3 fibroblast (non-labeled) and C6 glioma (GFP-labeled) cells are co-cultured at designated cell densities.

**Figure 4:**
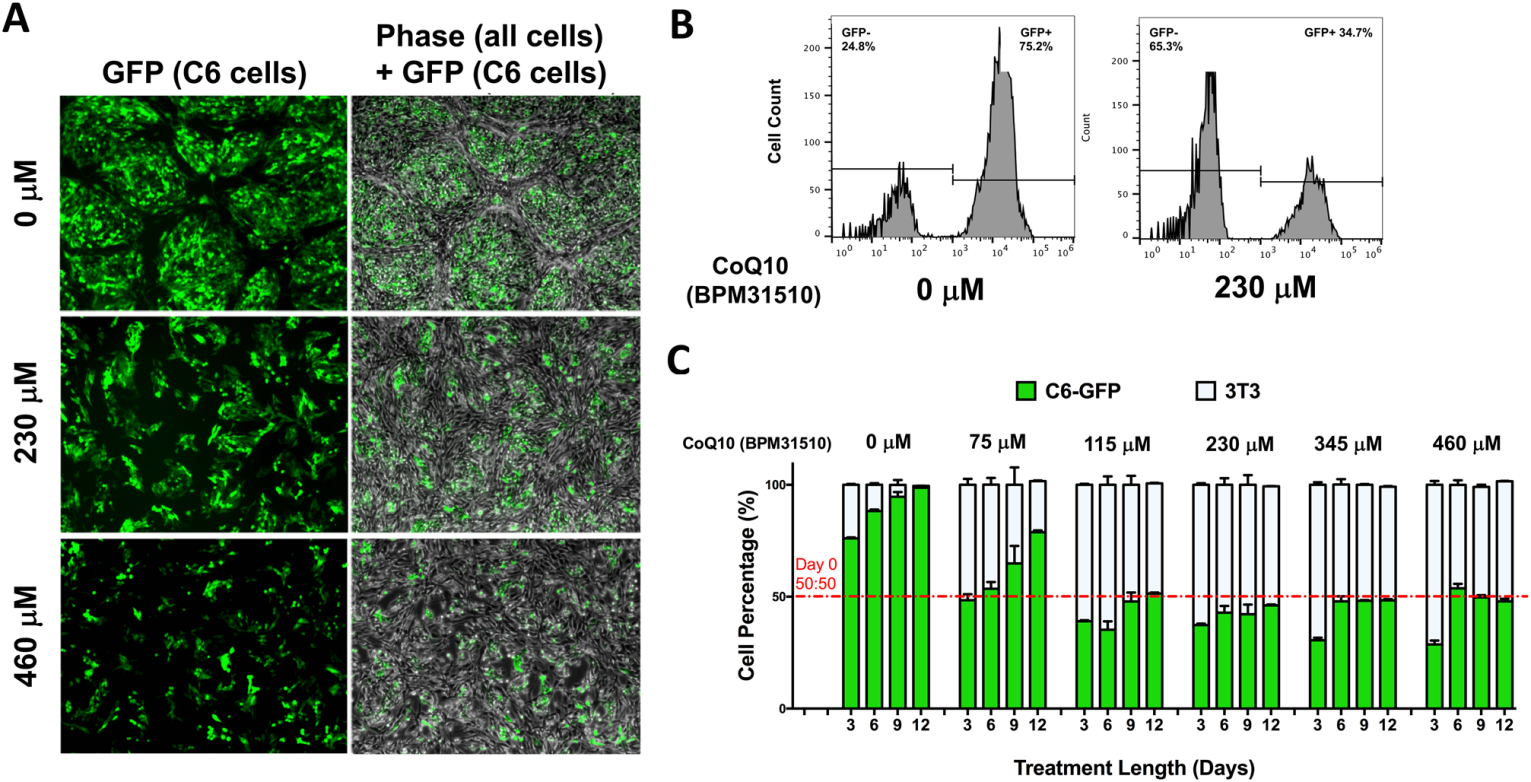
Ubidecarenone induces differential effects on cell growth and redox vulnerabilities between non-tumor and glioblastoma cells in co-culture. (**A**) Phase and fluorescent images of GFP-labeled rat C6 glioma cells and non-labeled mouse NIH3T3 fibroblast, co-cultured and treated with 0, 230, or 460 µM ubidecarenone, demonstrates a dose-dependent decrease in glioma cells with a relative sparing of HA cells. **(B**) Flow cytometry analysis (scatter plot) of GFP and O_2_^−^ in GFP-labeled rat C6 glioma cells and non-labeled mouse NIH3T3 co-culture. Cell populations are characterized based on GFP intensity. Note the increase in the GFP_neg_ population relative to GFP_high_ population in the presence of ubidecarenone. (**C**) Flow cytometry analysis of GFP-labeled rat C6 glioma cells and non-labeled mouse NIH3T3 fibroblasts co-cultures treated with ubidecarenone for up to 12 days. Cells are characterized based on their GFP intensity (GFP_neg_ or GFP_pos_). Results are grouped based on ubidecarenone dose and are shown as a percentage of the entire cell population. While glioma cells (GFP_pos_) represent the entire cell population by day 9 in co-cultures without ubidecarenone, there are essentially equal numbers of glioma and non-tumor cell populations at doses ≥ 115 µM ubidecarenone.

**Figure 5:**
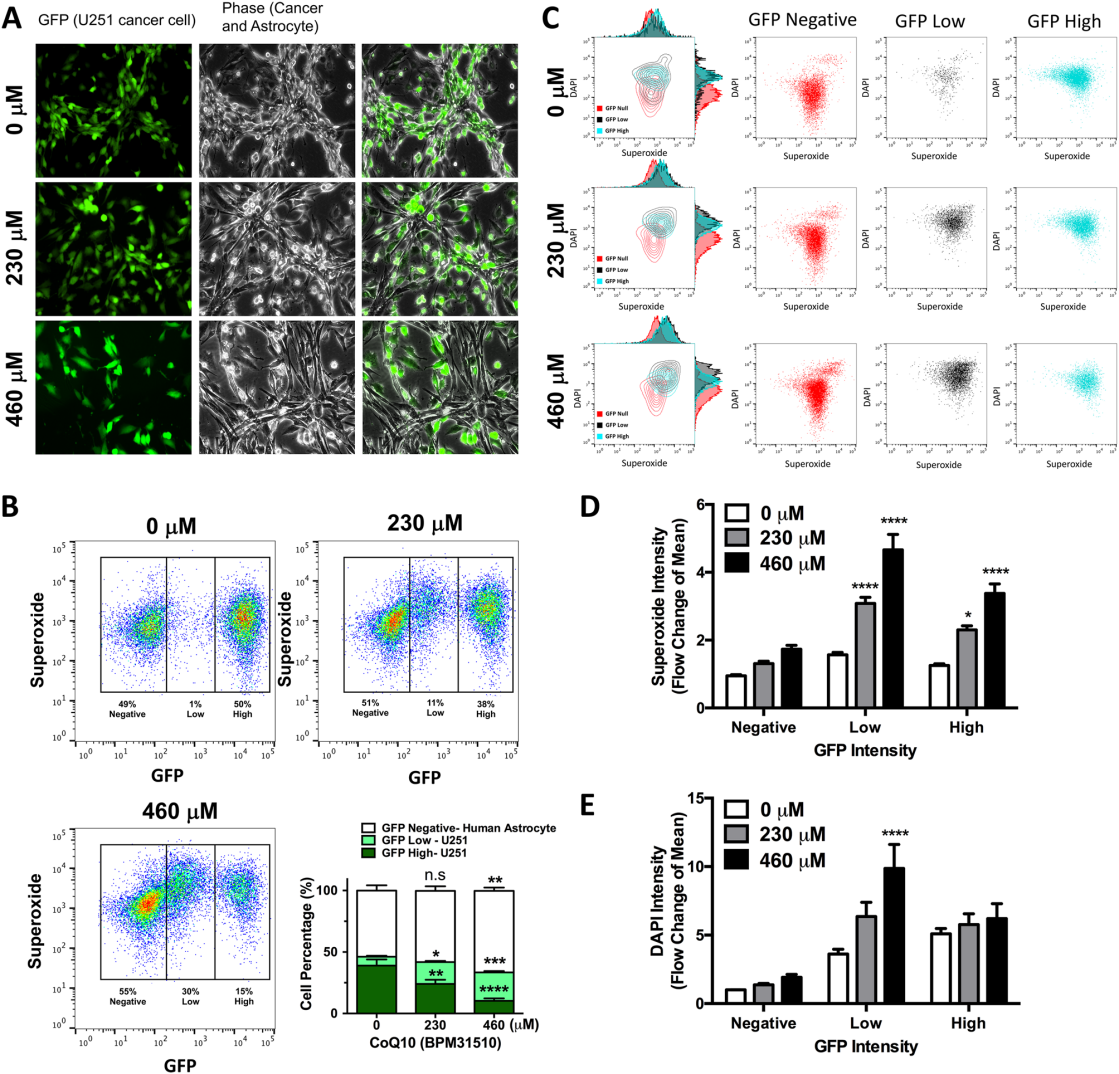
Ubidecarenone induces differential effects on cell growth and redox vulnerabilities between non-tumor and glioblastoma cells in co-culture experiments. (**A**) Phase and fluorescent images of GFP-labeled human U251 glioma cells and non-labeled HA cells co-cultured and treated with 0, 230, or 460 µM ubidecarenone demonstrate a dose-dependent decrease in glioma cells with a relative sparing of HA. **(B**) Flow cytometry analysis (scatter plot) of GFP and O_2_^-^ in GFP-labeled human U251 glioma cells and non-labeled HA cells in co-culture. Cell populations are characterized based on GFP intensity (GFP_neg_, GFP_low_, and GFP_high_). Note the dose-dependent increase in the GFP_neg_ population relative to the GFP_high_ population in the presence of ubidecarenone. Quantification of flow cytometry cell populations illustrates a dose-dependent increase in GFP_low_ cell numbers. (**C**) Flow cytometry analysis of O_2_^−^ and DAPI in GFP-labeled human U251 glioma cells and non-labeled HA cells, co-cultured and treated with 0, 230, or 460 µM ubidecarenone. Note the increase in O_2_^−^ values for both GFP_low_ and GFP_high_ cells, with insignificant changes noted in the GFP_neg_ (HA cells) population. (**D**) Graphical depiction of flow cytometry analysis of O_2_^−^ and DAPI in GFP-labeled human U251 glioma cells and non-labeled HA cells, co-cultured and treated with 0, 230, or 460 µM ubidecarenone. In contrast to the GFP_neg_ population, BPM exposure results in a marked increase in superoxide production in both the low and high GFP fractions. (**E**) DAPI levels are significantly elevated only in the GFP_low_ cell population, consistent with this population representing dying cells.

GFP-labeled rat C6 glioma cells (C6_GFP_) form clusters and surround non-labeled mouse NIH3T3 fibroblast cells after 72 h in co-culture under basal conditions (Fig. 4A). Co-culture incubation with 230 or 460 µM ubidecarenone induced a dose-dependent decrease in the distribution of C6_GFP_ cells in comparison to non-labeled NIH3T3 cells, which were less impacted by ubidecarenone (Fig. 4A). Graphically illustrated in Fig. 4B, 72 h after equal numbers of C6_GFP_ and non-labeled NIH3T3 cells were initially plated, 75% of the cell population was GFP labeled (C6_GFP_) in the absence of ubidecarenone compared to only 35% after incubation in 230 µM ubidecarenone. Of note, the inhibitory effects on the cancer cell population persisted. In cultures maintained up to 12 days without ubidecarenone, C6_GFP_ cells represented the entire cell population. In contrast, cultures with ubidecarenone doses above 115 µM were equilibrated and persisted for 12 days (Fig. 4C). Utilizing a similar co-culture strategy with GFP-labeled human glioma U251 and non-labeled HA cells, a robust response wherein increasing ubidecarenone doses differentially depleted GFP-labeled human glioma U251 cells in comparison to non-labeled HA cells was noted (Fig. 5A).

Next, flow cytometry was utilized to assess the differential effects on O_2_^−^ and DAPI staining when ubidecarenone doses are increased. Three distinct cell populations, GFP-negative, GFP-low, and GFP-high were noted (Fig. 5B). Given the unlikely event that HA cells acquired GFP, the GFP_low_ population is thus interpreted to represent human glioma U251 cells. Consistent with this, a dose-dependent accumulation of GFP_low_ human glioma U251 cells that correlated with a significant reduction in the GFP_high_ population was noted (Fig. 5B), implicating ubidecarenone exposure in this transition.

### Ubidecarenone exploits differential redox vulnerabilities between non-cancerous and glioblastoma cells

We next assessed each of the three cell populations (GFP_neg_, GFP_low_, and GFP_high_) to compare changes in O_2_^−^ and DAPI intensity, which occur after exposure to ubidecarenone (Fig. 5C). First, consistent with the known increase in basal oxidative stress, we noted that while O_2_^−^ levels for human glioma U251 cells were 1.5-fold higher than HA cells under basal conditions, the difference increased over fourfold after treatment with BPM31510 (Fig. 5D). These findings are consistent with previous observations in experiments conducted with independent cell lines (Fig. 3D) and support the contention that ubidecarenone exploits differential redox vulnerabilities between human glioma U251 cells and HA to mediate its anti-cancer activity. Consistent with knowledge regarding the Warburg effect, our findings further suggest that our system mimics reality.

Noteworthy, ubidecarenone did not induce significant changes in either O_2_^−^ production or DAPI staining in the non-labeled HA cells (GFP_neg_). In contrast, the GFP_low_ population exhibited a notable increase in both O_2_^−^ production and DAPI staining, while the GFP_high_ population exhibited a significant increase in O_2_^−^ levels only (Fig. 5C–E). Consistent with the cell cycle analysis, these findings suggest that the GFP_low_ population represents the actively dying fraction of tumor cells.

### Ubidecarenone exerts efficacy in an orthotopic model of glioblastoma

**Figure 6:**
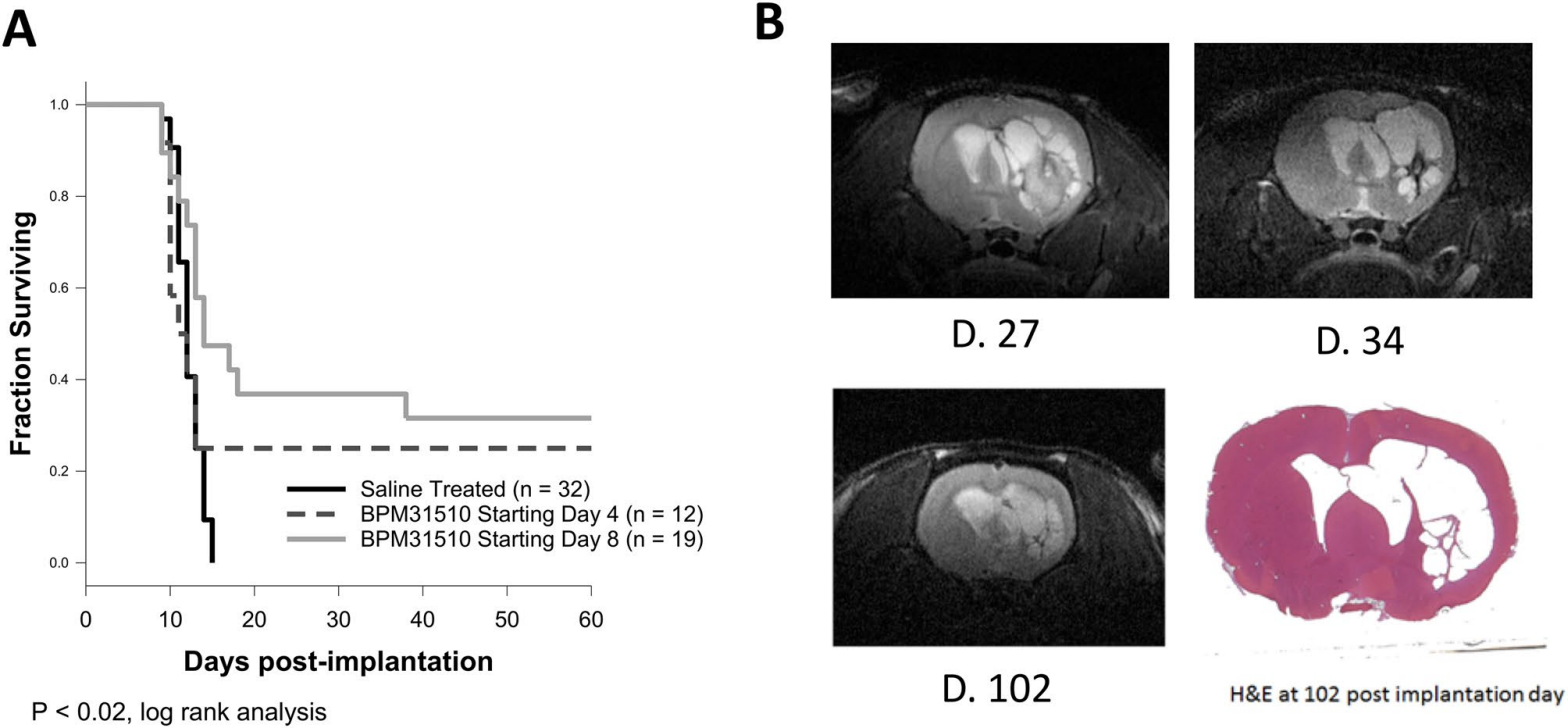
Ubidecarenone demonstrates efficacy in an orthotopic glioma model. Wistar rats received either saline or 50 mg/kg BPM31510 IP, twice daily, five days per week, starting at either 4 (n = 12) or 8 (n = 19) days after implantation of 10^6^ C6 glioma cells into the right striatum. (**A**) Survival of rats treated either with saline (n = 32) or BPM31510. Over 25% of BPM31510 treated rats survived to the end of experiment (at least 75 days) compared to 0% of PBS treated controls (P < 0.01, log rank statistic). No significant difference was noted between the BPM31510 treated groups starting at 4- or 8-days. (**B**) Serial MRI of a long-term survivor (Day 27, Day 34, and Day 102 post-implantation) demonstrating persistent effects even after treatment was withdrawn. Lower right panel is a coronal plane H&E stained section of the same long-term survivor demonstrating a cystic cavity with no obvious tumor present.

To address whether ubidecarenone exerts in vivo efficacy, we assessed its effects in an orthotopic model using rat C6 glioma cells. After inoculation of 10^6^ cells into the right striatum, control rats (n = 32) all died within 16 days of implantation. In contrast, nine of 31 (29%) rats, treated with BPM31510 50 mg/kg i.p. twice per day beginning either 4 or 8 days after implantation, and continuing through post-implantation days 35–42 were long-term survivors (P < 0.02, log rank statistic; Fig. 6A).

While median survival was increased modestly (median 12.0 vs. 13.0, saline vs. BPM31510, P < 0.01, log rank statistic), there was a marked increase in the number of rats surviving greater than 16 days (0% vs. 29%, p < 0.001, Fisher’s Exact test). Furthermore, gradual involution was noted over time in long-term survivors with the appearance of ex vacuo changes pathologically characterized as cystic cavities containing no macroscopic tumor over six weeks after drug cessation (Fig. 6B). No significant differences were noted relative to the start of treatment relative to tumor implantation.

## Discussion

Ubidecarenone delivered using BPM31510 at levels equivalent to those achieved using native Ubidecarenone has no appreciable effects on glioma cells; however, the increased solubility in the lipid nanodispersion formulation allows for increased exposure (> 200 fold). Utilizing cellular proliferation assays, cell cycle analysis, and measurements of mitochondrial O_2_^−^ production, even at ubidecarenone doses well below the maximum levels achievable, differential effects were observed in tumor cells relative to non-tumor cells. Furthermore, the aforementioned differential effects were maintained in co-culture experiments, with prolonged drug exposure resulting in an equilibrated cultures where neither tumor nor non-tumor cells dominated over time.

Malignant gliomas possess several features that make it a prototypical tumor type for novel metabolic approaches, including the presence of extensive metabolic reprogramming, a high level of oxidative stress, and its development within an environment that is at once relatively inaccessible and very sensitive to normal tissue damage. This was the impetus for the investigation into ubidecarenone’s efficacy both in vitro and in an orthotopic glioma model. In vitro, our results indicate that administration of BPM31510 results in a marked differential elevation in mitochondrial O_2_^−^ species in two established glioma cell lines compared to two non-tumor lines derived from both human and rat. This elevation in mitochondrial O_2_^−^ species preceded the onset of slowed growth and G2/M cell cycle arrest.

The noted slowed growth is of particular interest when comparing the effects of ubidecarenone in an immortalized 3T3 murine line and HA, the latter being almost completely insensitive to growth delay, and exhibiting markedly diminished elevations in O_2_^−^ production, even at high doses. Whether the marked resistance of HA relative to NIH3T3 cells to the effects of ubidecarenone reflects the fact that human cells express only CoQ_10,_ compared to the relative abundance of CoQ_9_ in rodents^19,20,38^, rather than reflecting a difference in sensitivity related to immortalized vs. non-immortalized cells, remains to be determined.

The co-culture studies are especially illuminating in how this therapy might result in a different, but equally efficacious, outcome compared to conventional cytotoxic approaches. The observation of equilibrated cultures which persisted over time suggest that modulating the redox status “leveled the playing field” between cancer and non-cancer cells (Fig. 4). Translating this to potential in situ observations, one might expect that optimal dosing redox balance could be achieved long term, resulting in a period of extended control without a marked impact on conventional anatomical imaging.

The idea of creating equilibrium through exerting differential redox toxicity is also of interest in light of the in vivo experimental results. The observation of an essentially “all or none” response characterized by either rapid death or a slow involution of established tumors is unusual and raises the question of why this agent does not result in a cure in all rats. While one could posit that variable penetration of drug to tumor tissue might underlie this variation, it is also very possible that ubidecarenone’s effectiveness is dependent on maintaining an optimal redox balance. Generally supporting this contention is accumulating evidence that raising ROS levels can selectively induce cancer cell death by disabling antioxidants^39,40^ only if ROS is sufficiently elevated to preferentially disable cancer cells relative to normal ones. Therefore, methods in which this effect can be measured in vivo are required if this strategy is to succeed in the clinic.

While a standardized in vivo measure of oxidation status in cancer tissues does not yet exist, a number of potential imaging methods might prove useful in this regard. For instance, measuring compensatory endogenous antioxidants such as glutathione could be envisioned as a readout, wherein the ratio of the oxidized and reduced forms would reflect redox status. Alternatively, PET tracers such as hydroascorbate, which can also provide key information about redox status^41,42^, are currently being studied.

In summary, we demonstrate that exposure to high levels of ubidecarenone produce differential changes in glioma cells relative to non-glioma cells. This effect correlates with the production of intramitochondrial O_2_^−^, an increase that is noted well before changes in proliferation or the cell cycle can be measured. Considering that non-tumor cells appear resistant to its growth inhibiting effects, including O_2_^−^ production, BPM31510 possesses attributes warranting further evaluation as a redox biologic agent.

## Methods

### Orthotopic C6 glioma model

1 × 10^6^ rat C6 glioma cells were injected intracranially into 6–8 week-old Wistar female rats as previously described^43^ with approval of the Stanford University School of Medicine IACUC (Protocol #11396). In brief, Wistar adult rats weighing 150 to 200 g were anesthetized initially with isofluane (3–4% followed by maintenance 1–2%) and placed in a stereotactic head holder. A burr hole approximately 3 mm lateral and posterior to the bregma using a 19-gauge dental drill was then performed. Suspensions of 10^6^ exponentially growing C6 glioma cells in 30 µl of MEM were injected over a 5-min period through a Hamilton syringe placed 3 mm lateral and 3 mm posterior to the bregma and 0.6 mm deep to the dura. Four and eight-days post-implantation, rats were randomly allocated to receive either isotonic PBS (n = 32) or 50 mg/kg bid BPM 31,510 starting either 4 (n = 12) or 8 days post-tumor implantation (n = 19). I.P. injections were continued for 5 days per week until rats met requirements for euthanasia (i.e., eye rings lethargy, motor incoordination) or day 35 post-implantation.

### Reagents

CoQ_10_ (C9538) and dimethyl formamide (DMF, 33120) were purchased from SIGMA-ALDRICH (St. Louis, MO). BPM31510 was prepared as previously described^34^ and provided by BERG LLC (Framingham, MA).

### Cell culture

GP2-293 cells (Catalog #. 631458) was obtained from CLONTECH, now TAKARA BIO USA (Mountain View, CA). Rat C6 GBM (Catalog# CCL-107), and mouse NIH3T3 fibroblasts (Catalog# CRL-1658) were obtained from ATCC (Manassas, VA). Human U251 GBM (Catalog# 03063001) was from SIGMA-ALDRICH (St. Louis, MO). All cells were maintained following manufacturer’s protocols. Normal human astrocytes (HA, Catalog# CC-2565) were purchased from LONZA (Basel, Switzerland) and grown in HA growth medium kit (Catalog# 821–500) from APPLICATION INC. (San Diego, CA). For the co-culture system experiments, labeled tumor and unlabeled non-tumor cells were seeded in 6-well plates at designated densities and treated with BPM31510 or vehicle, for 24–72 h. Rat C6 GBM and NIH3T3 fibroblast cells were co-cultured in DMEM medium supplemented with 10% FBS, and human U251 GBM and HA cells were co-cultured in HA growth medium.

### Retroviral production and establishment of stable GBM cell lines

GP2-293 cells were grown in a T75 flask dish to 85% confluence and transfected with pQCXIP-EGFP and pVSVG vector (Gifts from Dr. Nan Gao, Rutgers-Newark) using Lipofectamine 3,000 (Catalog# L3000015) from LIFE TECHNOLOGIES (Carlsbad, CA) following manufacturer’s protocol. The plasmid information was described at a previous paper^44^. Virus collection and cell infection steps were modified from a previous protocol^44^. Briefly, the medium containing the retroviruses was collected 48 h post-transfection, centrifuged at 3,000 rpm to remove cell debris, and the supernatant passed through a 0.45 µM filter. Glioma cells (80% confluence) were then incubated with viral-DMEM medium containing 40% filtered supernatant and 60% fresh DMEM with 10% FBS supplement for 6 h, followed by culture in DMEM medium with 10% FBS supplement for 48 h. Stable clones were selected by culturing with puromycin (1 μg/ml) for 7 days, followed by flow cytometry to purify the GFP-positive population.

### Cell viability assay and cell counts

Cell viability was evaluated by measuring the fluorescence signal generated from the cell viability reagent PrestoBlue following manufacturer’s instructions (Catalog# A13261) from THERMOFISHER (Waltham, MA). In brief, cells were plated in cell culture medium in a 96-well plate in triplicate at 5,000 cells/well and incubated with indicated concentration of compounds. Cell viability are assessed after 24, 48 or 72 h of treatment by fluorometer analysis (Excitation/ Emission (nm) is 560/595) using Prestoblue assay. All data is presented as Mean ± SEM of three replicated experiments.

Cell numbers for human U251 GBM, rat C6 GBM, mouse NIH3T3 fibroblasts, and normal human astrocytes were calculated based on individual standard curves generated from known numbers (ranged from 5,000 to 600,000 cells per well) of each cell line.

### Cell cycle analysis

10^6^ cells were fixed and permeabilized with 70% ethanol for 30 min at − 20 °C. After permeabilization, cells were washed twice with cold PBS and pelleted. Cells were then resuspended in 300–500 µL FxCycle PI/RNase staining solution (Catalog# F10797) from INVITROGEN (Carlsbad, CA). Cells were subjected to flow cytometry after incubation for 30 min in the dark. The percentage of cells in each phase (G_0/1_, S, or G_2_ + M) was then estimated from the frequency histograms.

### MitoSOX-based O_2_^−^ staining

Cells were cultured in 6-well plates with designated reagents and incubated according to experimental design. For flow cytometry measurements, cells were gently twice-washed with 37 °C pre-warmed Hank’s Balanced Salt Solution (HBSS, Catalog# 14175079) from THERMOFISHER (Waltham, MA) and then incubated in the dark with 5 µM MitoSOX working solution (Catalog# M36008) from THERMOFISHER (Waltham, MA) for 10 min at 37 °C. Cells were then harvested from the plates and washed twice with 37 °C pre-warmed HBSS. A final concentration of 3 µM DAPI dye (Catalog# 62248) from THERMOFISHER (Waltham, MA) was then added into each sample to distinguish live/dead cells. The cell samples were then analyzed by flow cytometry to determine the mean fluorescence intensity and percentage of stained cells.

### Flow cytometry

Flow cytometry data were obtained from a Scanford instrument and the transfected GFP-positive glioma cells were sorted using a Megatron instrument in the Shared FACS Facility at Stanford University.

### Microscopic analysis

Microscopic images were obtained using a Leica CTR5000 microscope and MetaMorph software program. Images were processed using ImageJ.

### Quantification and statistical analysis

All data were analyzed from indicated independent experiments. Cell viability or fold change in cell numbers were averaged from six independent wells in each independent experiment, and each experimental condition was repeated three or more times. Data were plotted as mean ± SEM. FACS experiments were independently repeated in triplicate and analyzed using Flowjo. Statistical analysis was performed using two-way ANOVAs on the basis of experimental setups for cell cycle analysis and percentage of cell populations, or one-way ANOVA on the basis of experimental setups for cell viability, fold change in cell numbers, and mean of O_2_^-^ or DAPI. Graphs were constructed with GraphPad Prism 5 and results represented graphically as *P < 0.05; **P < 0.01; ***P < 0.001 or ****P < 0.0001.

## Supplementary information

Supplementary file1

## Data Availability

All data supporting the findings of this study are available with the article and can also be obtained from the authors.
