# Supplementary material for: High levels of ubidecarenone (oxidized CoQ10) delivered using a drug-lipid conjugate nanodispersion (BPM31510) differentially affect redox status and growth in malignant glioma versus non-tumor cells
Source: Sci Rep. 2020 Aug 17;10:13899. doi: 10.1038/s41598-020-70969-0 (PMC7431533; doi:10.1038/s41598-020-70969-0)
Supplement: Supplementary file 1 — Supplementary file1 [file 41598_2020_70969_MOESM1_ESM.pdf]

Supplementary Information

High levels of ubidecarenone (oxidized CoQ_{10}) delivered using a drug-lipid conjugate nanodispersion (BPM31510™) differentially affect redox status and growth in malignant glioma versus non-tumor cells

Jiaxin Sun PhD[1,*], Chirag Patel MD, PhD[1], Taichang Jang PhD[1], Milton Merchant MS[1], Chen Chen PhD[2], Shiva Kazerounian, PhD[3], Anne R. Diers, PhD[3], Michael A. Kiebish, PhD[3], Vivek K. Vishnudas, PhD[3], Stephane Gesta, PhD[3], Rangaprasad Sarangarajan, PhD[3], Niven R. Narain, PhD[3], Seema Nagpal MD[1], Lawrence Recht MD[1,*]

[1]Department of Neurology and Clinical Neurosciences, Stanford University, Palo Alto, CA 94305

[2]Department of Otolaryngology, Stanford University, Palo Alto, CA 94305

[3]BERG LLC Framingham, MA 01701

[*]Correspondence: Jiaxin Sun (JIAXSUN@STANFORD.EDU), Lawrence Recht (LRECHT@STANFORD.EDU)

Supplementary Figures 1-4

Supplementary Figure Legends

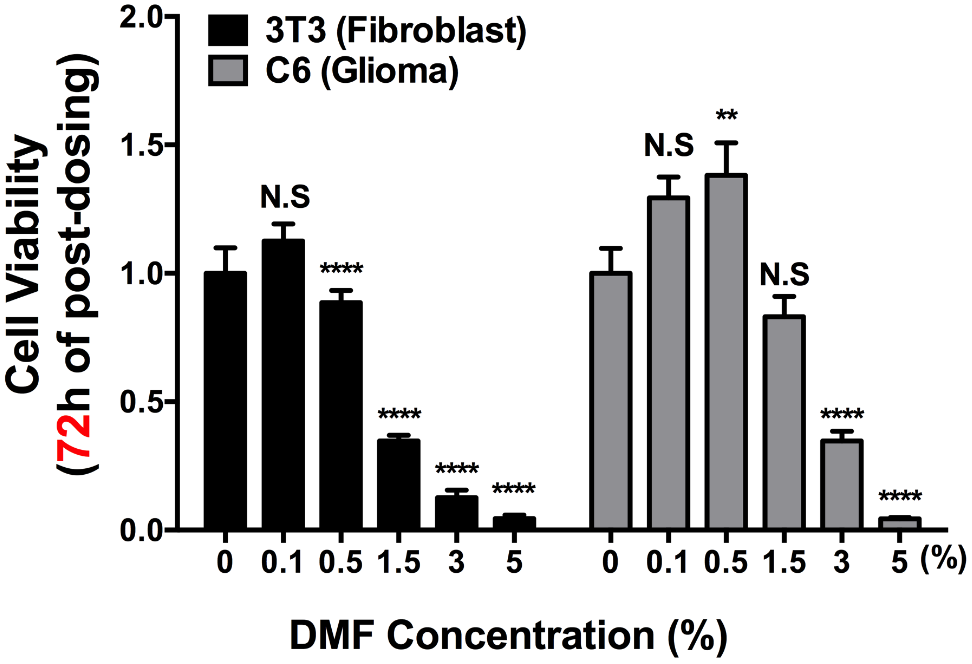

Sun et al. Supplementary Figure 1

Figure S1. Cytotoxic effects of dimethylformamide (DMF) on rodent NIH3T3 fibroblasts or rodent glioma C6 cells

Cell viability analyses of rodent NIH3T3 and rodent glioma C6 cells were performed to study the inhibitory effects of dimethylformamide (DMF). Relative cell viability was measured utilizing presto blue assay and cells were treated with 0, 0.1, 0.5, 1.5, and 3.5% DMF, respectively. All data are presented as Mean± SEM and **P < 0.01; ****P < 0.0001; compared to the control (no DMF).

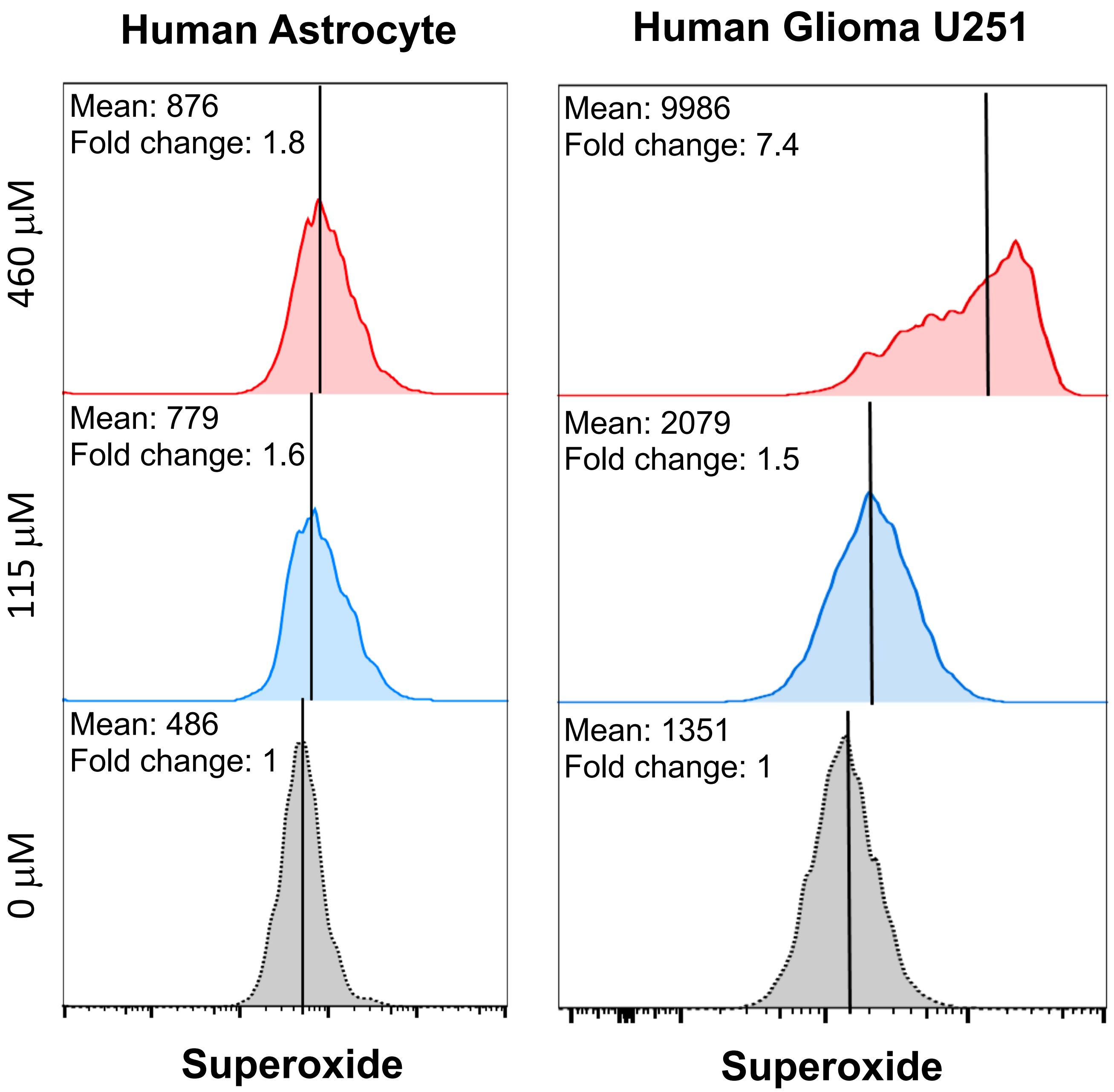

Sun et al. Supplementary Figure 2

Figure S2. O_2^- intensity in human glioma U251 and HA cells

Flow cytometry frequency histograms of O_2^- for HA and human glioma U251 cells are demonstrated. Mean values were computed using Flowjo software, and fold changes were calculated and normalized to the control (0 μM exposure) using the same cell line. Note the marked fold change (7.4) in human glioma U251 cells compared to HA (1.8) at highest dose.

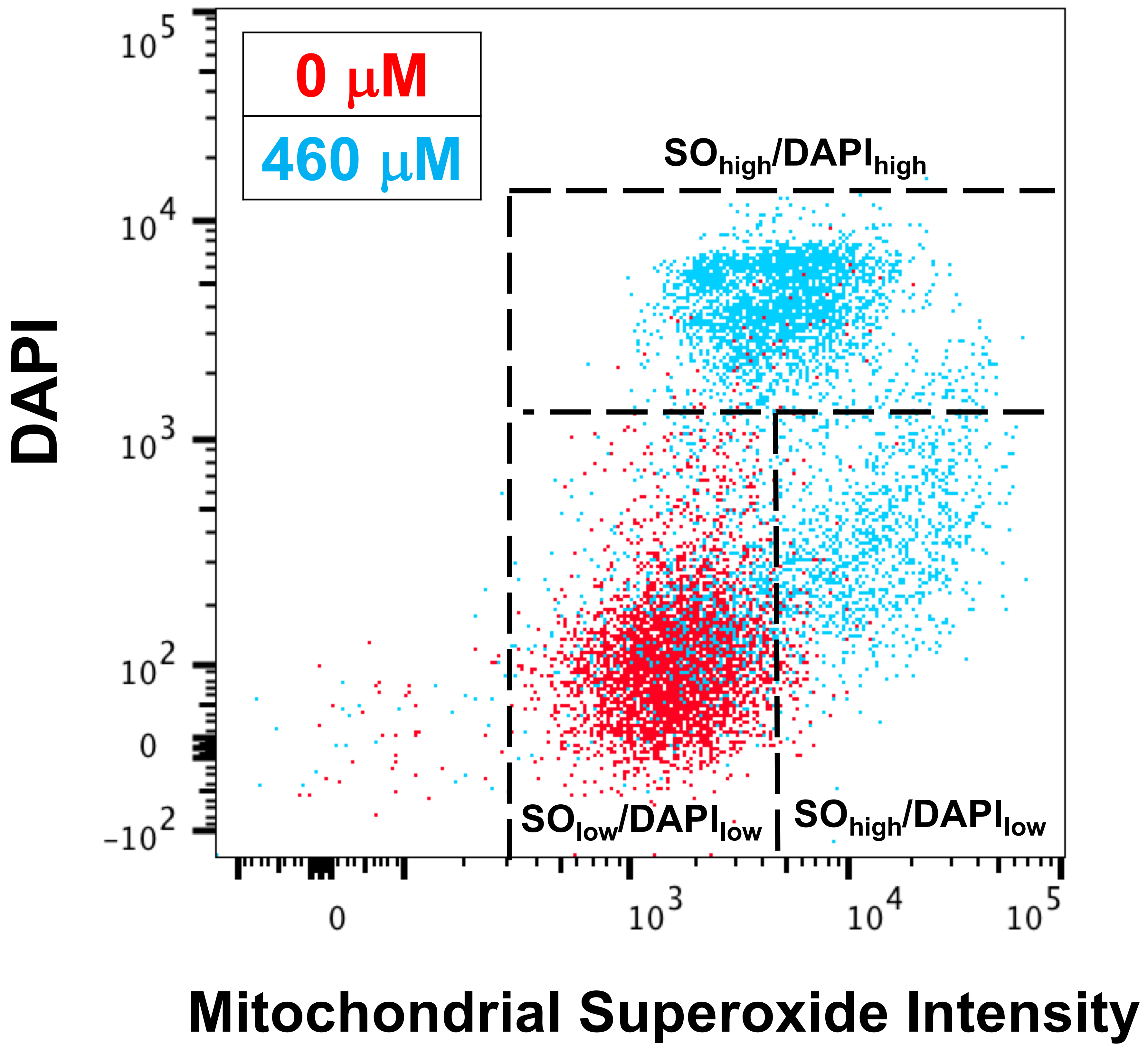

Sun et al. Supplementary Figure 3

Figure S3. Example of distinct populations emerging within the glioma cell population

Based on DAPI and O_2^- intensity, cells were characterized into three populations ($O_2^-{}_{low}$/$DAPI_{low}$, $O_2^-{}_{high}$/$DAPI_{low}$, and $O_2^-{}_{high}$/$DAPI_{high}$).

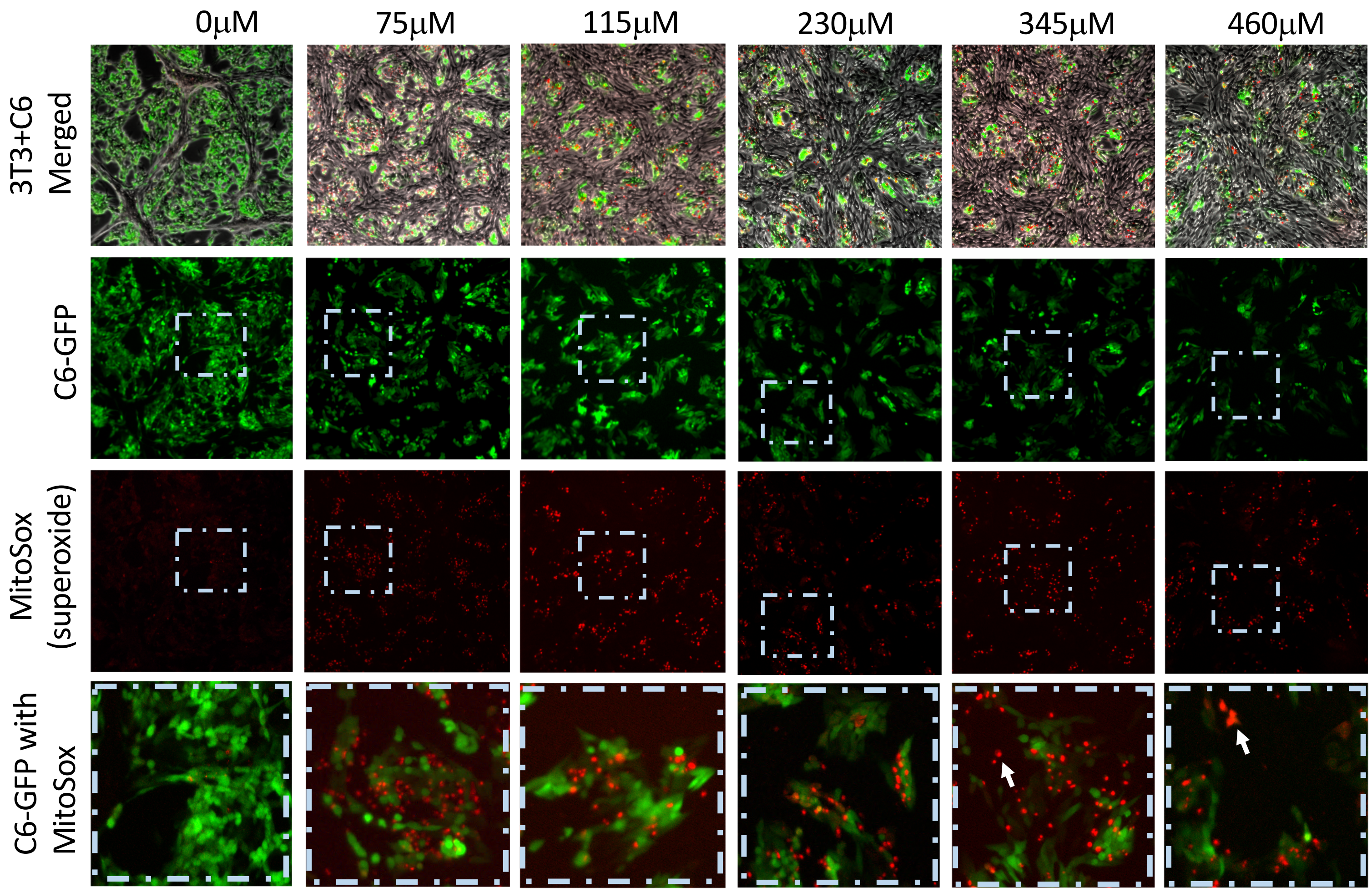

Sun et al. Supplementary Figure 4

Figure S4. Differential induction of mitochondrial superoxide by BPM31510 in non-cancer vs. neoplastic cells.

NIH3T3 and C6 cells were initially seeded in 1:1 ratio. After 72h incubation with BPM31510 (0µM, 72µM, 115µM, 230µM, 345µM or 460µM), images were obtained using a Leica CTR5000 microscope. Superoxide levels are visualized using Mitosox staining. Images demonstrate high co-expression of active superoxide (red) in the C6 (GFP) population, whereas superoxide production by NIH3T3 cell (no fluorescent label) is only noted at 345µM or 460µM (white arrows).
